# Cerebrospinal fluid production rate in various pathological conditions: a preliminary study

**DOI:** 10.1007/s00701-023-05650-2

**Published:** 2023-06-24

**Authors:** Kanza Tariq, Ahmed Toma, Sogha Khawari, Meriem Amarouche, Mohamed A. Elborady, Lewis Thorne, Laurence Watkins

**Affiliations:** 1 National Hospital for Neurology and Neurosurgery, Queen Square, London, UK; 2 St George’s Hospital, Blackshaw Road, London, UK

**Keywords:** Cerebrospinal fluid production rate, Hypersecretion, Hydrocephalus, Liquoguard7

## Abstract

**Introduction:**

The cerebrospinal fluid (CSF) production rate in humans is not clearly defined but is estimated to be 18–24 ml/h (Trevisi et al Croat Med J 55(4):377–387 (24); Casey and Vries Childs Nerv Syst 5(5):332–334 (8)). A frequent clinical observation is that patients often drain higher volumes of CSF than can be explained by the assumed ‘normal’ CSF production rate (PRcsf). In the National Hospital for Neurology and Neurosurgery PRcsf was recorded in a variety of common neurosurgical pathologies using LiquoGuard7, an automated peristaltic pump that accurately controls CSF drainage and maintains a pre-set CSF pressure.

**Methods:**

A prospective observational study was performed from September 2021 onwards, on all patients in the National Hospital for Neurology and Neurosurgery who required CSF drainage as part of their ongoing treatment. The external drain was connected to a LiquoGuard7 pump (Möller Medical GmbH, Fulda, Germany), and the internal software of LiquoGuard7 was used to measure PRcsf. Statistical analysis used SPSS (version 25.0, IBM) by paired *t* test, comparing measured rates to hypothetical ‘normal’ CSF production rates calculated and published by Ekstedt (16–34ml/h) (Ekstedt J Neurol Neurosurg Psychiatry 41(4):345–353 (14)), assuming a similar distribution.

**Results:**

PRcsf was calculated in 164 patients. Suspected normal pressure hydrocephalus (*n*=41): PRcsf of 79ml/h±20SD (*p*<0.0001). Post-surgical CSF leak (*n*=26): PRcsf of 90ml/h±20SD (*p*<0.0001). Subarachnoid haemorrhage (*n*=34): PRcsf of 143ml/h±9SD (*p*<0.0001). Intracerebral haemorrhage (*n*=22): PRcsf of 137ml/h±20SD (*p*<0.0001). Spinal lesions (*n*=7): PRcsf of 130ml/h±20SD (*p*<0.0032). Pituitary adenomas (*n*=10): PRcsf of 29 ml/h±9SD (*p*<0.049). Idiopathic intracranial hypertension (*n*=15): PRcsf of 86ml/h±10SD (*p*<0.0001). Decompensated long-standing overt ventriculomegaly (*n*=4): PRcsf of 65ml/h±10SD (*p*<0.0001). Cerebral infection (*n*=5): PRcsf of 90ml/h±20SD (*p*<0.0001).

**Conclusion:**

Net CSF production rate may be higher than expected in many conditions, as measured with new device LiquoGuard7 through the study of net flow rate, which may have implications for clinical decisions on CSF diversion. The conventional understanding of CSF production and circulation does not explain the findings of this study. More extensive studies are needed to validate this technique.

## Introduction

Historically cerebrospinal fluid (CSF) is believed to be formed at a rate of 18 – 24 ml/h, renewed approximately 3–5 times a day, maintaining a total CSF volume of 90–150mls and a supine ICP between 7 and 15mmHg [Trevisi G, Frassanito P, Di Rocco C (2014)^8^, ^24^]. However, a frequent clinical observation is that patients often drain higher volumes of CSF than can be explained by the assumed normal CSF production rate.

Hydrocephalus is thought to be predominantly due to obstruction in CSF flow and only rarely due to CSF overproduction by choroid plexus hypertrophy, infection or tumour [9, 12]. Still, idiopathic CSF over-secretion has been recognised since 1989 [8]. Trevisi et al. reported a case of post-operative CSF leak and IIH in which a CSF production rate (PRcsf) of approximately four times the normal was calculated through daily measurement of externally drained CSF through an externalised shunt [24], and almost 2-fold increase has been discovered in some instances of normal pressure hydrocephalus (NPH) [5, 16]. Phase-contrast magnetic resonance imaging (PC-MRI) has also detected CSF hyperdynamic flow in cases of Chiari I and syringomyelia [5] Similarly, almost fourfold increase in PRcsf was calculated and reported by Bauer et al. in a cases of meningitis and cerebral infection [3] through daily measurement of CSF as collected by a ventriculostomy.

Recent studies employing PC-MRI, radioactive water and biological dyes have challenged the traditional views of CSF hydrodynamics. A significant volume of CSF is formed by the choroid plexus and the remaining amount by the brain parenchyma, ependyma and the blood capillaries constituting the blood-brain barrier (BBB) [6, 15, 17]. It is absorbed by the arachnoid villi , as well as by the nasal lymphatics via the perineural spaces along the cribriform plate. Macromolecules and waste products are also removed along the intramural and perivascular pathways of the brain vasculature and probably by the dural lymphatics. The exchange of CSF between the interstitial fluid and perivascular spaces is called the glymphatic system [4, 15, 17].

CSF production by the choroid plexus is mediated through apical and basolateral membrane transporters such as aquaporin 1 (AQP1), K^+^/Cl^−^ co-transporter, Na^+^/K^+^ ATPase and glucose transporter GLUT1 [6]. At the BBB, the blood-CSF barrier of the arachnoid membrane, Virchow-Robin (VRS) space, brain parenchyma and ependyma, alongside ion transporters, water transport is mediated through aquaporin 4 (AQP4), which are located in the end-feet of astrocytes [6].

AQP1 and AQP4 are both associated with normal CSF hydrodynamics but are also implicated in several pathologies. AQP4 is associated with the development and resorption of cytogenic and vasogenic cerebral oedema respectively [4, 6, 24]. It is also over-expressed in hydrocephalus, traumatic brain injury, brain abscess, meningitis, cortical freeze-injury, hypoxic injury and ischemia [6, 11, 24]. Absence of AQP4 is associated with lack of clearance of beta-amyloid from the central nervous system, consequently leading to Alzheimer’s disease [4]. Both AQP4 and AQP1 are significantly upregulated in cerebral injury, haemorrhage and tumours [6].

Experiments studying the role of transporters in CSF production found > 20% reduction in CSF production in AQP1 null mice, and 7–10 fold decreased CSF production in AQP4 null mice [6], although this may be more pronounced in the condition of IIH [11]. CSF production has also been observed to be altered by change in the ventricular CSF osmolarity compared to serum osmolarity [7].

Previously CSF was thought to maintain a unidirectional bulk flow driven by the hydrostatic gradient generated by the choroid plexus and the arachnoid villi. PC-MRI has revealed an additional pulsatile flow in synchrony with the cardiac and respiratory cycles [7, 17, 20]. Investigations employing neuroimaging and biological dyes suggest that the continuous exchange between CSF, blood and interstitial fluid is likely to contribute to much higher CSF production rates than previously assumed [6, 17, 20].

Similarly, studies into the impact of barbiturate anaesthesia on CSF flow revealed that PRcsf is approximately 6.5 times less in an anaesthetised animal as compared to an awake animal [15]. Studies with fluorescent markers also revealed 14% CSF flow rates in awake states in animals as compared to 23% in sleep and highlighted the role of sleep in brain re-generation through the role of CSF hydrodynamics in metabolite clearance [6, 15, 26]. This transition in flow rates between asleep and awake states took approximately 15 min. Therefore, the results of earlier experiments into the calculation of PRcsf may have been misleading because the experiments were conducted in anaesthetised animals.

Over the decades, several techniques have been employed to calculate PRcsf. Since CSF is simultaneously secreted and absorbed, a production rate was estimated based on the absorption rate of CSF, calculated from choroid plexus arterial blood flow and haematocrit value [6]. Pressure studies following lumbar drainage, ventriculo-cisternal perfusion and rate of cisterna magna marker removal were used to calculate PRcsf, but were deemed unreliable and impractical in clinical settings due to invasiveness [6, 15, 21].

We measured PRcsf and intracranial pressure (ICP) in patients with various neurosurgical pathologies, using LiquoGuard7, an automated device certified for simultaneous monitoring of pressure and precise drainage of CSF [10]. It was hypothesised that multiple pathologies are associated with a rise in PRcsf, and the study was aimed at defining to which degree CSF production rate differs from the traditional estimates in the respective ailments. This observation would have implications for prognosis and would be a paradigm shift in our understanding of CSF physiology.

The LiquoGuard pump is already in clinical use for CSF drainage and early mobilisation of patients [1], ICP monitoring [18] and prevention of spinal cord ischemia during thoracoabdominal aortic surgery [19, 25], but its use for PRcsf calculation is novel although based on accepted techniques using lumbar puncture and manometry.

## Methodology

In 1977 and 1978 Ekstedt published his studies of CSF hydrodynamics in humans, employing constant pressure infusion based on similar techniques used by Davson et al. for animal studies in 1970 [13, 14]. By constant infusion of artificial CSF at a pressure below cerebral venous pressure, he was able to eliminate CSF absorption by the arachnoid villi, and was able to collect CSF at its formation rate through a lumbar drain [13, 14].

Electron microscopic examination of the hydrostatic bulk flow through the arachnoid villi exhibited a pressure-dependant one-way vacuolation of pores [6]. Absorption of CSF into the venous system occurs when CSF pressure is higher than the cerebral intravenous pressure and is therefore dependent upon intracranial pressure. Confocal microscopy and immunohistochemical studies show that this is also true of fluid transport across pial membranes and CSF absorption into the venous plexus of the nasal mucosa [6].

Therefore, decreasing CSF pressure below cerebral venous pressure halts CSF absorption into the venous circulation. Under such circumstances, CSF collected through external drainage devices represents the basal CSF production.

Transporters at the BBB and the blood-CSF barrier create an osmotic gradient which contributes to CSF production and absorption at these barriers [6, 15]. Even if it is assumed that lowing CSF pressure below cerebral venous pressure does not stop CSF re-absorption through the osmotic channels, the studied PRcsf calculation represents the net CSF flow rate in these neurosurgical conditions.

### Calculation of the net PRcsf

A PRcsf study was performed on all patients in our centre who required CSF drainage through an extra-ventricular drain (EVD) or a lumbar drain (LD), as part of their ongoing clinical management from September 2021 onwards.

EVD (Codman® Bactiseal®, Integra LifeSciences, USA) was inserted through Kocher’s or Keen’s point depending on individual cases. LD (Medtronic® Duet epidural catheter, USA) was inserted between L3/L4 lumbar vertebrae. The drainage catheters in all cases were attached to LiquoGuard7. The external tubing of LiquoGuard7 apparatus was primed with 0.9% saline to prevent wastage of CSF.

The LiquoGuard7 pump has a pressure transducer, which measures the CSF pressure relative to that reference point. According to published studies in a completely supine individual the external auditory meatus (EAM) correlates to the anatomical brain centre and provides a suitable zero level for CSF pressure measurement [23].

**Fig. 1:**
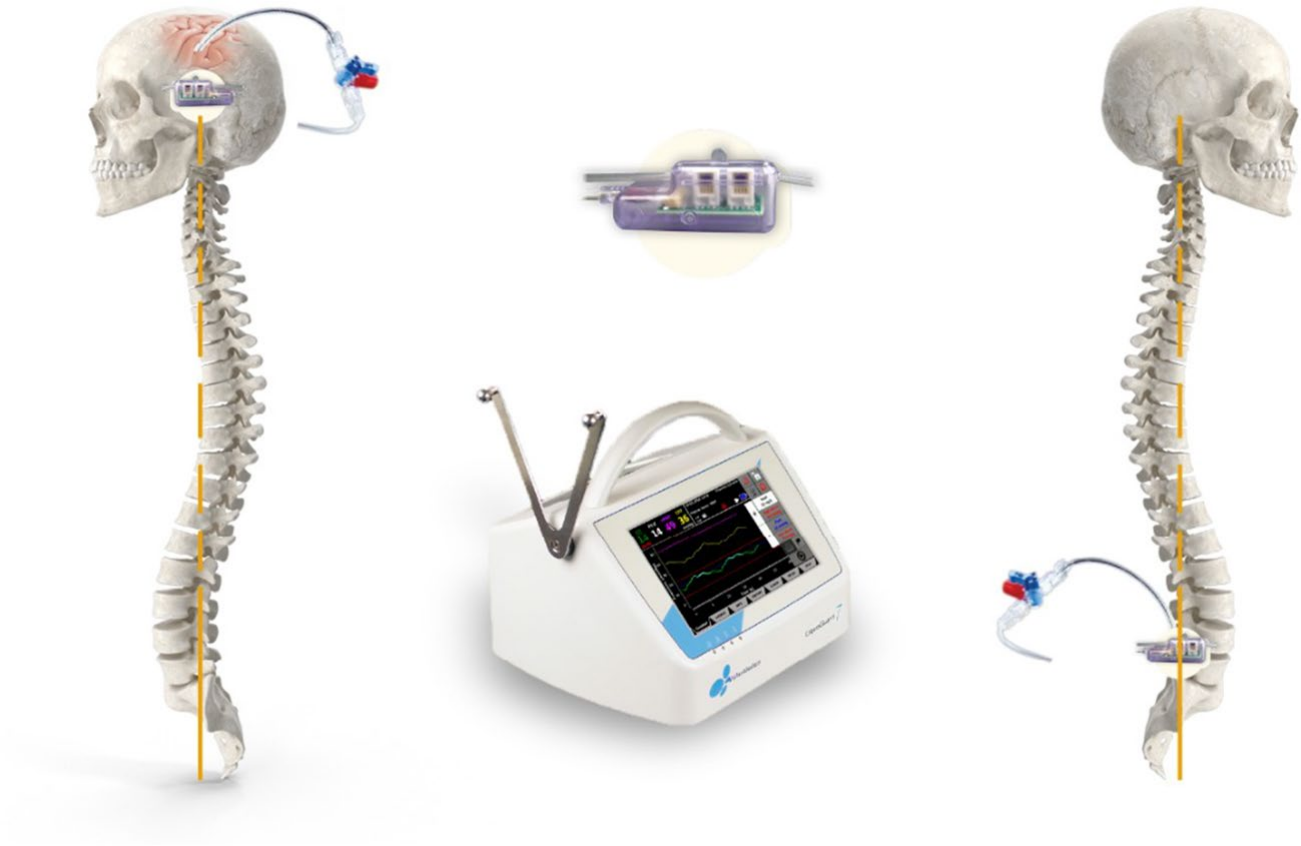
LiquoGuard7 external ICP transducer applied at the level of the external auditory meatus in patients requiring CSF drainage through an extra-ventricular drain or a lumbar drain

For this study the ICP transducer was applied on the skin of the patients at the level of the EAM with an electrocardiogram sticker provided with the LiquoGuard7 drainage set (Fig. 1).

**Fig. 2:**
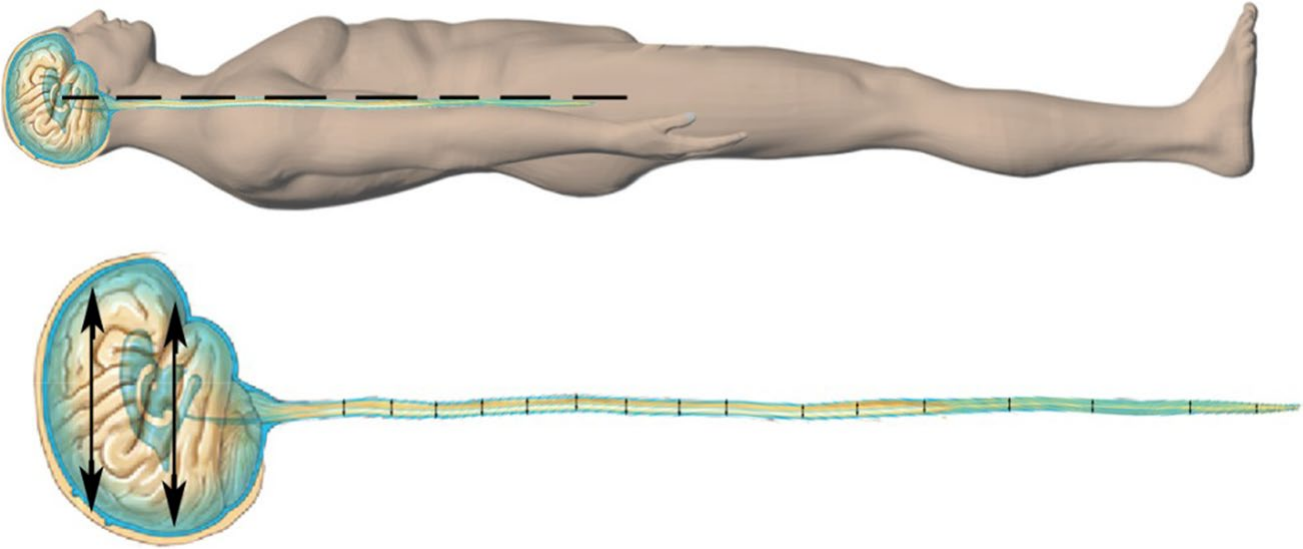
In a completely supine position the CSF pressure is uniform in all the fluid compartments of the central nervous system

The patients were requested to lie in a flat, supine position. Only patients who were able to safely lie flat for the duration of the study were included. In a flat, supine position the CSF pressure is uniform in all the fluid compartments of the central nervous system, allowing for accurate CSF pressure measurement by the external ICP sensor applied at the level of the EAM [22] (Fig. 2).

The LiquoGuard pump allows for two modes of drainage: volume-led drainage and pressure-led drainage. For this study, pressure-led drainage was employed. This mode allows for CSF removal and collection in the external collecting bag as soon as the ICP exceeds the set pressure limit. For the purpose of the study the pressure limit of the machine was set at 0mmHg, which is sufficiently low to prevent CSF absorption into the venous circulation.

With CSF pressure set at 0 mmHg, the volume of the fluid which was collected into the external collection bag attached to the LiquoGuard7 was set at 150ml/h, which is the upper limit of the LiquoGuard7. This also represented a short-coming of the current study, as it prevents from recording PRcsf above 150ml/hour.

**Graph 1:**
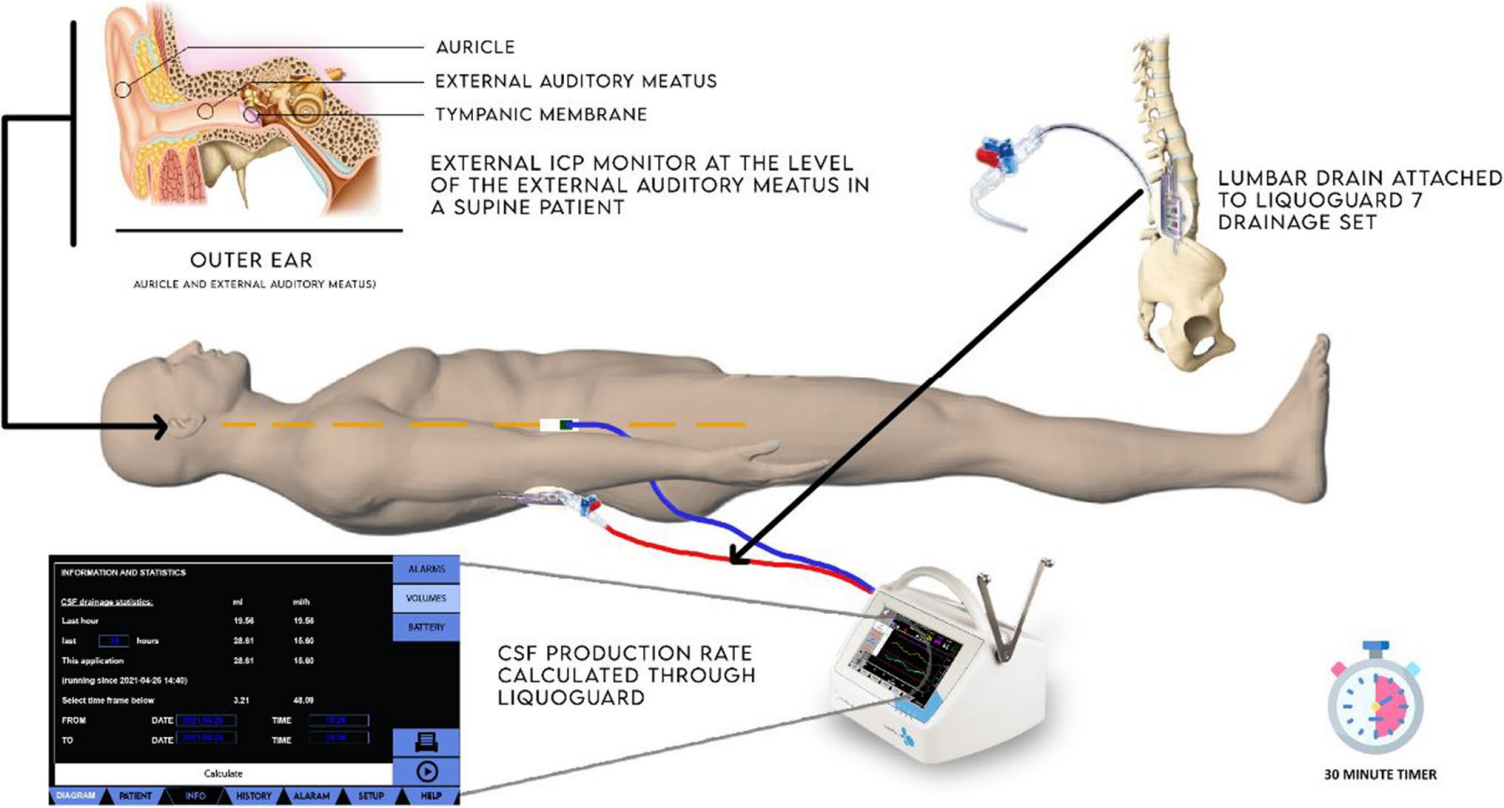
Showing the ICP before the initiation of the study, the second-to-second variation in ICP during a typical 30-min study session, and ICP after the study

During initial studies, it was established that in most supine patient with the above mentioned test parameters, net CSF flow rates stabilised to a constant value within 4 to 15 min. What this means is that in a strictly supine individual connected to a LiquoGuard7 with the external transducer at the level of EAM, when the CSF pressure is set at 0mmHg and the upper limit of volume to be drained is set at 150ml/h, the LiquoGuard7 machine attempts to remove as much CSF as it can, as much as 150ml/h, to try to bring the ICP to 0. Since the patient is strictly supine this ICP is representative of the CSF pressure of the entire central nervous system (CNS) in a neutral position. Hence, during initial studies it was observed that with the above mentioned test parameters the LiquoGuard7 drained out CSF at high flow rates in an attempt to bring CSF pressure to 0, and that this flow rate progressively decreased with each minute as ICP decreased, until the CSF pressure reached a value of 0 and the flow rate stopped decreasing any further but instead remained the same from that time-point onwards. CSF pressure increased above 0 within seconds reflecting release of CSF in the CNS, and was almost instantly brought back to 0 by LiquoGuard7 via draining the released CSF, the quantity of which could be calculated by the internal software of LiquoGuard7 pump and remained constantly stable (Graph 1).

The second-to-second variation in CSF pressure could also be seen in the ICP waveform displayed by the LiquoGuard7. It was found that the stabilisation of CSF flow rate took between 4 and 15 min. Subsequently, the study protocol was refined to allow for 20 min for the net CSF flow rate to stabilise to a constant. Consequently, the patients were requested to remain supine for a minimum duration of 50 min: 20 min for the net CSF flow rates to stabilise and a further 30 min for the calculation of net CSF production rate. Net CSF production rate was calculated after each 10 min for a total of 30 min and was found to be constant in the whole number with variations only in the decimal number. ICP was simultaneously continuously studied, with consideration paid to ICP before the net CSF flow rate measurement, ICP during the net CSF flow rate measurement and ICP immediately after the net PRcsf study.

**Graph 2:**
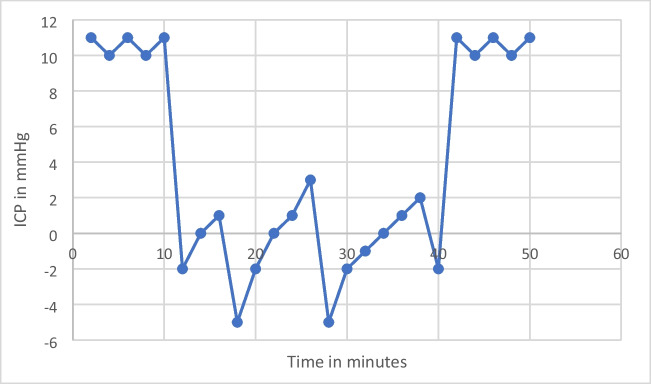
CSF flow rate as seen during a typical study session in NPH patients. CSF flow rate stabilises in 4 to 15 min in most patients and then remains constant for the duration of the study (30 min)

The LiquoGuard7 software can record flow rate data collected over 30 min and calculate an hourly CSF production rate. The LiquoGuard7 software is licenced for accurate flow rate data recording (Graph 2).

The study was repeated on 3 consecutive days for each patient, at the same time each day to avoid diurnal variations in net basal CSF production.

Other CSF diversion devices in situ and the settings of these devices were noted.

When the patient is completely supine with CSF pressure at 0mmHg, a normally functioning differential pressure shunt does not allow for any CSF to be drained from the ventricles, since the intra-abdominal pressure will always be greater than 0mmHg.

When patients were undergoing prolonged external CSF drainage for clinical reasons, the study was repeated after an interval of 4 weeks, to access changes in net CSF production rate with time.

Seven patients required a LD after the removal of the EVD. Net CSF flow rates were calculated in these patients from both cranial and lumbar sites.

**Fig. 3:**
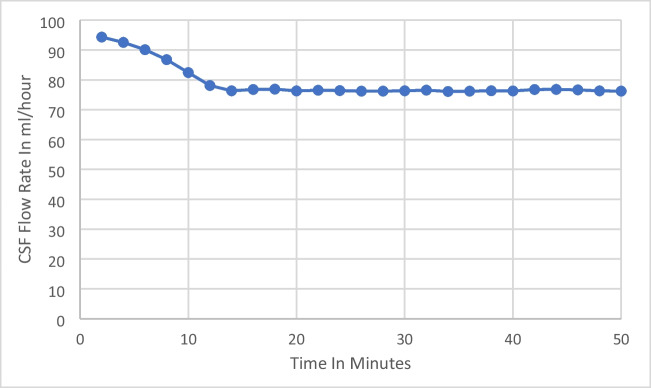
In a completely supine patient requiring CSF drainage through a LiquoGuard7, the external ICP transducer was placed on the skin at the level of the external auditory meatus. The LiquoGuard7 drainage parameters were set at pressure-led 0mmHg and drainage volume of 150ml/h. Patients were requested to remain flat for 30 min of CSF production rate study using LiquoGuard flow rate data

Patients were followed up for 6 months. Patient outcome on the Glasgow Outcome Scale was compared with the early measurements of net PRcsf (Fig. 3).

### Statistical analysis

Statistical analysis was conducted by performing a paired *t* test comparing the measured PRcsf to the assumed ‘normal’ CSF production rates published by Ekstedt in 1978 and believed to be between 16 and 34ml/h [14], using statical software SPSS (version 25.0, IBM).

A multivariate linear regression analysis was performed to evaluate the impact of any other in situ CSF diversion devices on the PRcsf.

## Results

One hundred and sixty four patients with various neurosurgical disorders were studied prospectively from September 2021 onwards. Extended CSF drainage was accomplished through an EVD connected to LiquoGuard7 in 64 patients and a LD in 100 patients. Seven patients required a LD after the removal of the EVD. Twelve patients had pre-existing ventriculoperitoneal shunts (VPS) with programmable valves in situ. All patients with a particular diagnosis had similar results, regardless of age, gender or co-morbidities. A brief summary of each neurosurgical disorder examined is given below (Table 1).

**Table 1:** Demographic data of the studied patients

Demographical data	
Number of patients	164
Gender (M:F)*	53:111
Average age	59
Age range	20–87
EVD	64
LD	100
Both EVD and LD	7
Programmable shunts in situ	12

*Male:female

### Normal pressure hydrocephalus and long-standing overt ventriculomegaly

CSF production rate was calculated in 41 normal pressure hydrocephalus (NPH) and 4 decompensated long-standing overt ventriculomegaly (LOVA) patients via extended lumbar drainage through LiquoGuard7 (Table 2).

**Table 2:** Data for CSF production rate in normal pressure hydrocephalus and decompensated longstanding overt ventriculomegaly

Disease studied	Number of patients	Male:female	Average age	Average net PRcsf	*p* value	ICP before the study	ICP during study	ICP after the study	Pre-assessment CSF diversion devices in situ	Post-assessment CSF diversion devices in situ
NPH	41	22:19	77	55–82ml/h	(*p*<0.0001)	7–11	−5 to 2	7–11	0	38
LOVA	4	1:3	55	58–79ml/h	(*p*<0.0001)	5–12	−2 to 3	5–12	0	4

Twenty-two NPH patients demonstrated an average net PRcsf of 55–82 ml/h (*p*<0.0001). Of these, two NPH patients with previous spinal pathology had average net PRcsf of 69–92ml/h. At a 6-month follow-up all 22 patients had received a programmable VPS and exhibited a Glasgow outcome scale (GOS) of 5 with improved neurological, psychological and motor status.

Seventeen suspected NPH patients had an average net PRcsf of 22–32ml/h (*p*=2.13). With clinical investigations these patients failed the diagnosis of NPH and were non-responsive to extended lumbar drainage. At a 6-month follow-up these patients had a GOS of 4 with ongoing cognitive and motor deficit.

Two NPH patients were observed to have an average net PRcsf of 22–32ml/h (*p*=2.13), but showed subjective and objective symptomatic improvement with ELD. At a 6-month follow-up these patients had received a programmable VPS and exhibited a Glasgow outcome scale (GOS) of 5 with progressive improvement in neurological, psychological and motor function.

Average net PRcsf in the 4 LOVA patients was found to be 58–79ml/h. At 6-month follow-up all 4 patients had received a programmable VPS and exhibited GOS of 5.

### Idiopathic intracranial hypertension

In our study patients with IIH (*n*=15, LD) displayed an average net PRcsf of 83–94ml/h (*p*<0.0001) (Table 3).

**Table 3:** Data for CSF production rate in idiopathic intracranial hypertension

Disease studied	Number of patients	Male:female	Average age	Average net PRcsf	*p* value	ICP before the study	ICP during study	ICP after the study	Pre-assessment CSF diversion devices in situ	Post-assessment CSF diversion devices in situ
IIH	15	1:14	36	83–94ml/h	(*p*<0.0001)	9–15	−5 to 6	9–15	5	11

Five of the studied patients had a pre-existing programmable VPS but presented with acute visual deterioration following shunt malfunction.

Five patients underwent Superior Sagittal Sinus (SSS) stent insertion. Their net PRcsf post-stenting was calculated to be average 130–150ml/h . Eleven patients eventually received CSF diversion and had a 6-month GOS of 5.

### Spontaneous and post-operative CSF leakage

Patients with spontaneous or post-operative CSF leakage had an average net PRcsf of 75–110ml/ (*p*<0.0001) (Table 4).

**Table 4:** Data for CSF production rate in CSF leak

Disease studied	Number of patients	Male:female	Average age	Average net PRcsf	*p* value	ICP before the study	ICP during study	ICP after the study	Pre-assessment CSF diversion devices in situ	Post-assessment CSF diversion devices in situ
CSF leak	26	11:15	51	75–110ml/h	(*p*<0.0001)	7–15	−2 to 8	7–15	0	19

At six-month follow-up, 19 out of the studied 26 patients had received permanent CSF diversion and were symptom-free with GOS 5.

### Spinal lesions

Net PRcsf was assessed in six intradural extramedullary spinal tumours and one traumatic spinal fracture, via a LD. The average net PRcsf in all six patients was 100–150ml/h (*p*<0.0001). At 6-month follow-up four patients had received a shunt and had a GOS of 5 (Table 5).

**Table 5:** Data for CSF production rate in spinal lesions

Disease studied	Number of patients	Male:female	Average age	Average net PRcsf	*p* value	ICP before the study	ICP during study	ICP after the study	Pre-assessment CSF diversion devices in situ	Post-assessment CSF diversion devices in situ
Spinal lesions	7	4:3	42	100–150ml/h	(*p*<0.0001)	8–16	−1 to 5	8–16	0	4

### Subarachnoid haemorrhage and intracerebral haemorrhage

Net PRcsf was assessed in 34 SAH patients and 22 ICH patients requiring CSF drainage through an EVD (Table 6).

**Table 6:** Data for CSF production rate in subarachnoid haemorrhage and intracerebral haemorrhage

Disease studied	Number of patients	Male:female	Average age	Average net PRcsf	*p* value	ICP before the study	ICP during study	ICP after the study	Pre-assessment CSF diversion devices in situ	Post-assessment CSF diversion devices in situ
SAH	34	14:20	59	135–150ml/h	(*p*<0.0001)	7–22	−2 to 7	7–22	7	17
ICH	22	12:10	58	120–150ml/h	(*p*<0.0001)	8–25	−5 to 11	8–25	0	12

Seven of the patients with SAH had a pre-existing programmable shunt. The average net PRcsf in 19 SAH patients and 13 ICH patients was above 120ml/h, with the mode of the cohort being 150ml/h (*p*<0.0001).

Extended lumbar drainage was required in seven SAH patients following the removal of EVD. Net PRcsf was re-examined in these patients through LD and was found to be the same as measured from EVD site in the whole number with variations only in the decimal number.

Four patients required extended CSF drainage via an EVD for over a month. In these patients net PRcsf was re-calculated following an interval of 4 weeks from the initial study. It was found to be the same as before i.e. between 120 and 150ml/h.

At 6-month review, 17 of the SAH patients, and 12 of the ICH patients had received VPS insertion and had a GOS of 5.

Fifteen SAH patients and nine ICH patients demonstrated a net PRcsf of 20–25ml/h, much lower than most of the other patients in these groups. All these ICH patients and twelve of these SAH patients had a GOS 1 within weeks of the bleed. Three SAH patient had GOS of 3 at 6-month follow-up.

### Cerebral infection

Net CSF flow rate was studied in two patient with micro-abscesses, two patients with ventriculitis and one patient with cerebritis requiring CSF drainage through an EVD. The net average CSF production rate was found to be 77–106ml/h (*p*<0.0001) (Table 7).

**Table 7:** Data for CSF production rate in cerebral infection

Disease studied	Number of patients	Male:female	Average age	Average net PRcsf	*p* value	ICP before the study	ICP during study	ICP after the study	Pre-assessment CSF diversion devices in situ	Post-assessment CSF diversion devices in situ
Cerebral infection	5	0:5	51	77–106ml/h	(*p*<0.0001)	5–13	−4 to 5	5–13	0	4

At 6-month review, four of these patients had received VPS insertion and showed marked neurological improvement.

### Pituitary adenoma

Pituitary adenoma patients requiring CSF drainage through a LD demonstrated average net PRcsf of 26–36ml/h (*p*=0.059). At 6-month follow-up these patients had GOS of 5 and had returned to their baseline with no further intervention (Table 8).

**Table 8:** Data for CSF production rate in pituitary adenoma

Disease studied	Number of patients	Male:female	Average age	Average net PRcsf	*p* value	ICP before the study	ICP during study	ICP after the study	Pre-assessment CSF diversion devices in situ	Post-assessment CSF diversion devices in situ
Pituitary adenoma	10	1:4	44	26–36ml/h	(*p*<0.059)	4–11	−10 to 2	4–11	0	0

## Discussion

In these preliminary observations, several trends of raised CSF production associated with clinical need for early CSF shunting have been noted. These trends are in-keeping with similar incidental observations reported by others over the decades, in which CSF production rate was calculated by different methods without specifying the CSF pressure or the posture of the patient. Yet, the results observed in each of the mentioned pathology are comparable to those calculated in this study.

In two patients who underwent lumbar drainage following the removal of EVD, the PRcsf remained the same and did not vary between cranial and lumbar sites of CSF removal. This can be explained by the supine positioning of the patient during measurements, which makes CSF pressure uniform throughout all fluid compartments of the central nervous system.

Similarly, multiple CSF production rates calculated in the same patient 1 month apart yielded the same reading. This effect has also been reported by Casey et al. in a patient who was observed to produce four times the ‘normal’ amount of CSF months after suffering from a cerebral infection [8]. These findings can be explained by Hladky and Barrand, based on the works of James et al. (1977), Welch (1975) and Bering (1963), who concluded that PRcsf, once stabilised, does not change rapidly [15]. This could be attributed to the regulation of aquaporins in various pathological conditions [4, 6].

Aquaporin upregulation takes place in two phases. In an acute phase, aquaporin membrane channel permeability increases within seconds to minutes. In the second phase, long-term upregulation is maintained by changes in the rate of aquaporin mRNA synthesis within a few hours to several days [6]. This exerts a sustained effect on CSF production.

It is observed that relatively low or normal net PRcsf is a predictor of poor outcome in haemorrhagic stroke. Failure to maintain reflex hypersecretion of CSF seems to be associated with poor prognosis. CSF production rate may act as a prognostic tool in cerebral haemorrhage and other neurosurgical pathologies. This will be considered in depth in subsequent publication.

Stenting in IIH led to increase in net CSF flow rate. This may be because stenting increases the blood flow through the SSS, decreases the venous pressure and may lead to a change in the hydrostatic gradient resulting from increased secretion of CSF and increased absorption.

CSF production rate also appears to be a possible diagnostic tool in several common neurosurgical conditions such as NPH. In the studied cohort, patients who did not exhibit raised PRcsf were lumbar drainage non-responsive. This is significant since predicting shunt responsiveness in the context of NPH is still a clinical challenge [17]. Bradley et al., using PC-MRI, emphasised a PRcsf of ≥ twice the normal as a diagnostic parameter for NPH [5, 17]. These single centre observations also suggest net PRcsf as a diagnostic tool for NPH, although validation will be necessary in larger patient numbers, and will be discussed in detail in subsequent publication.

A history of previous spinal disease was noticed to be associated with increased net CSF flow rate in most patient groups.

Age, gender and non-spinal co-morbidities did not have any impact on the net PRcsf of any group, a fact also noted by Ekstedt during his experiments [13, 14].

It was also observed that at 6-month follow-up several patients with raised net PRcsf showed clinical improvement following the insertion of shunt. This is in keeping with previous studies exploring the role of intervention in the improvement of symptoms in patients in various diseases such as NPH and CSF leakage [2, 27].

Based on the individual net PRcsf observation of post-surgical spinal CSF leak patients, tailored CSF drainage of volume 50–150ml/h, continuously for a week, was offered to several patients. The individually personalised CSF drainage led to rapid recovery, prevention of significant complications and shorter hospital stay of the patients. This will be discussed in detail in subsequent publication.

Although the principle of this study was based on Ekstedt’s experiments on CSF hydrodynamics, LiquoGuard7 provides several technical advantages [13]. The technology does not require the pressure to be maintained externally through water columns in a manometer, as maintaining CSF pressure at a pre-set value is a function of the LiquoGuard7 system. No infusion was required; therefore, there was no need for any special infusion solution. None of the studied patients suffered any untoward side-effects such as headaches or leakage from the drain site. The methodology was also less invasive since it required no additional invasive procedures that were not already part of the patient’s clinical management.

This study helps provide a new understanding of the association of CSF production rate in these pathologies and to form a clinical decision support guide allowing for earlier decisions on CSF diversion, to provide optimal treatment, potentially preventing brain and spinal cord injury. This represents a paradigm shift in our understanding of hydrocephalus, which has generally been understood as obstruction of flow or absorption of CSF rather than an increase in CSF production. It may also open the possibility of new pharmacological targets for disease management, since increased production is more likely to be amenable to modification through a molecular approach than obstruction which has to be overcome by physical means.

## Conclusion

Net CSF production rate may be higher than expected in many conditions, as measured with new device LiquoGuard7 through the study of net flow rate, which may have implications for clinical decisions on CSF diversion. The conventional understanding of CSF production and circulation does not explain the findings of this study. More extensive studies are needed to validate this technique and to define its use as a diagnostic and prognostic method.
